# Localized provoked vulvodynia as an immune-mediated inflammatory disease: rationale for a new line of research

**DOI:** 10.3389/fcimb.2024.1505845

**Published:** 2024-12-17

**Authors:** Jorma Paavonen, Robert C. Brunham

**Affiliations:** ^1^ Faculty of Medicine, University of Helsinki, Helsinki, Finland; ^2^ Department of Medicine, University of British Columbia, Vancouver, BC, Canada

**Keywords:** vulvodynia, localized provoked vulvodynia, vulvar vestibulitis syndrome, vestibulodynia, inflammatory disease

## Abstract

Localized provoked vulvodynia (LPV), also called vulvar vestibulitis or provoked vestibulodynia, is a major cause of dyspareunia that severely impacts sexual health. At the tissue level, lymphocytic inflammation and hyperinnervation are characteristic pathological features, explaining the main symptoms and signs. A recent experimental animal study suggests that the histopathological findings of LPV may be due to mucosal CD4 Th17 immune responses to microbial antigens. We hypothesize that LPV is an immune-mediated inflammatory disease and challenge the concept of LPV as a chronic pain syndrome of unknown cause. Since most treatment modalities currently used in LPV are no better than placebo, we therefore warrant future research investigating the possible presence of CD4 Th17 cells and IL17 cytokine in affected tissues together with treatment trials that include inhibitors of the IL17 pathway.

Localized provoked vulvodynia (LPV) is defined as vulvar pain induced by touching the vulvar vestibular epithelium that has lasted 3 months or more, in the absence of any other recognizable causes of vulvar disease (Bornstein et al., 2016; ACOG & ASCCP, 2016). LPV is an important quality-of-life problem among young fertile-aged women with a peak incidence between 20 to 30 years of age. LPV is a problematic pain condition since the exact cause is unknown. At the tissue level, lymphocytic inflammation and neuron axonal proliferation and hyperinnervation are characteristic pathological features (Tommola et al., 2015, 2016). Most therapeutic approaches only alleviate symptoms and relapse is frequent. Most treatment modalities are no better than placebo (Miranda Varella Pereira et al., 2018). Systematic reviews and meta-analyses on different aspects of vulvodynia uniformly conclude that heterogeneity is the major limitation of current studies. In particular, high-quality, randomized controlled trials (RCTs) on LPV are needed.

By definition, LPV is characterized by moderate to severe pain upon light touch of the vestibular epithelium. Patients with LPV often react to localized vestibular pain with guarding and contraction of the pelvic levator muscle group making vaginal penetration difficult and painful. The clinical diagnostic test known as the Q-tip test, is performed using a simple cotton swab to gently touch the vestibular epithelium. The painful and inflamed areas are located around the vestibular glands, posteriorly and anteriorly. Cervical and vaginal infections or other dermatological conditions causing vulvar pain should be excluded before diagnosing LPV.

The etiology and pathogenesis of LPV is unknown. Women with LPV often have a history of recurrent vulvovaginal candidiasis (Leusink et al., 2018). Women with LPV also commonly have cutaneous hypersensitivity to *Candida albicans* (Ramirez De Knott et al., 2005). Vestibular fibroblasts from LPV patients produce more proinflammatory cytokines when stimulated with Candida antigens compared to fibroblasts from healthy control women (Foster et al., 2015). Also, fibroblasts from LPV patients express higher levels of dectin-1 fungal antigen receptor and respond to lower concentrations of Candida antigen than do fibroblasts from control subjects (Falsetta et al., 2015). Thus, microbial antigens from *C. albicans* can induce a long-lasting inflammatory response in the vulvar vestibulum. It is possible that other microbial or environmental antigens can trigger LPV in addition to Candida.

The histopathology of the vulvar vestibulum in LPV demonstrates infiltration with lymphocytic inflammatory cells and neuron axonal proliferation with hyperinnervation of sensory nerve fibers (Tommola et al., 2015, 2016). In areas with the highest density of lymphocytic inflammation, lymphoid aggregates represent tertiary lymphoid structures with increased density of B cells, activated plasma cells, and increased density of intraepithelial nerve fibers (Tommola et al., 2016, 2015). The proliferation of sensory nerve fibers detects mechanical pain, thus explaining the hypersensitivity to touch in LPV. Chronic inflammation may initiate hyperinnervation that causes allodynia (Tomalty et al., 2023). The VuNet-Vulvodynia Network project of a total of 1183 vulvodynia patients assessed comorbidities. Factors associated with vulvar pain included a high family history of diabetes mellitus, recurrent vulvovaginal candidiasis, urinary tract infections, irritable bowel syndrome, migraine, menstrual headaches, allergies, anxiety, and dysmenorrhea (Graziottin et al., 2020).

A systematic review of randomized controlled trials or non-randomized studies with a control group of the treatment of LPV including pharmacological, surgical, psychosocial, physiotherapeutic or combined interventions showed that most studies had low levels of evidence for effectiveness based on heterogeneity of interventions, poor outcome measures and inadequate comparisons (Bohm-Starke et al., 2022). The authors emphasize need for stringent RCTs and uniform outcome measures. Most studies have been underpowered and failed to demonstrate beneficial effects of an intervention. Research evidence on the efficacy of topical medications in LPV is similarly weak, although many such treatment modalities are commonly used in clinical practice. For instance, RCTs have not shown benefit from topical lidocaine or topical corticosteroids for alleviation of pain. Since chronic or recurrent vulvovaginal candidiasis is a potential risk factor for LPV, long-lasting maintenance therapy with antimycotics is commonly recommended. However, fluconazole use alone failed to demonstrate a significant benefit in LPV as reported in a RCT (Bornstein et al., 2000).

Oral antidepressants, mostly tricyclics or gabapentinoids are commonly used for generalized unprovoked vulvodynia. However, current evidence does not support antidepressants or anticonvulsant therapy in the treatment of LPV (Spoelstra et al., 2013).

Posterior vestibulectomy is one of the proven treatments that is effective for severe LPV. In clinical practice surgery is recommended for patients with severe LPV refractory to all conservative treatment attempts used. Overall, long-term patient satisfaction is strikingly high after surgery. Approximately 80-90% of the patients reported complete or partial response (Tommola et al., 2011). Of the surgically treated patients, 85% were satisfied with the treatment process, compared to 52% in the conservative treatment group. In another study, patients who underwent vestibulectomy between 1991 and 2003 were systematically interviewed. Of 85 eligible patients, 50 were contacted and 32 participated. Overall, 94% of those were highly satisfied, 97% would have surgery again, and 100% would recommend surgery to others (David and Bornstein, 2020). However, RCTs on the effectiveness of surgery have not been performed.

We believe that the major reason for the failure to find consistently effective therapy for LPV is the absence of understanding of its fundamental pathogenesis. We propose that LPV is an immune-mediated inflammatory disease due to CD4 Th17 immunopathology. A recent experimental animal model study showed that immunity to the microbiota promotes sensory neuron regeneration (Enamorado et al., 2023). Mice challenged intraepithelially with *Staphylococcus aureus* reproduced distinctive pathology with lymphocytic inflammation together with sensory neuron axonal proliferation. Specifically, the investigators demonstrated that CD4 Th17 cells accumulated beneath the infected epithelial surface around dermal nerves at the site of injury lead to neuron axonal proliferation in an IL17 dependent manner. Thus, IL17 acted as a major determinant of this fundamental process. Since this immune response was not protective in nature, it could incite immunopathology if unregulated. Because these experiments reproduce characteristic histopathology, we propose that CD4 Th17 responses to microbial antigens in vestibular epithelium can incite the pathophysiology of LPV (Majumder and McGeachy, 2021; Iliev et al., 2023). If this conjecture is correct, LPV joins a list of other related immune mediated inflammatory disorders. These are a heterogenous group of diseases such as rheumatoid arthritis, Crohn’s disease, ulcerative colitis, ankylosing spondylitis and psoriatic arthritis (Schett et al., 2021). Importantly, RCTs have demonstrated that several of these conditions are responsive to monoclonal antibody inhibitors of the IL17 system.

Our hypothesis suggests two lines of future investigation for this enigmatic LPV condition. These investigations are case-control studies of immunohistological analysis of lymphocytes infiltrating vestibular tissue from LPV patients and controls for CD4 Th17 cell markers and the demonstration of IL17 in affected tissue. If so, clinical trials with IL17 inhibitors should be undertaken. These drugs are already clinically used in the successful treatment of other immune-mediated inflammatory diseases such as psoriasis, psoriatic arthritis, lichen planus, and ankylosing spondylitis. Therapeutic targeting of individual cytokines or cytokine networks in immune-mediated inflammatory diseases such as the IL17 pathway has the potential for a mechanism-based intervention for LPV that avoids the need for surgical therapy. Randomized treatment trials of LPV are important since current therapies are not effective and patients with LPV often feel abandoned by the health care system.

## Data Availability

The original contributions presented in the study are included in the article/supplementary material. Further inquiries can be directed to the corresponding author.
